# A ICFEp Realmente Existe? Das Imitações à Classificação Errônea na Prática Clínica

**DOI:** 10.36660/abc.20250379

**Published:** 2026-05-11

**Authors:** Maria Giulia Bellicini

**Affiliations:** 1 ASST Spedali Civili di Brescia Brescia Itália ASST Spedali Civili di Brescia, Brescia – Itália

**Keywords:** Insuficiência Cardíaca Diastólica, Dispneia, Doenças das Valvas Cardíacas

Caro Editor,

Li com interesse o artigo recente de Villacorta et al.^1^ que aborda a distinção entre insuficiência cardíaca com fração de ejeção preservada (ICFEp) "verdadeira" e condições que apenas a imitam. Seu esforço para esclarecer essa ambiguidade conceitual é louvável e oportuno. No entanto, acredito que a questão se estende ainda mais: mesmo quando imitadores da ICFEp, como amiloidose, pericardite constritiva ou doença valvar grave, são cuidadosamente excluídos, a entidade clínica conhecida como ICFEp frequentemente se dissolve sob um exame mais detalhado.

Nos últimos 18 meses, realizei uma análise retrospectiva de 773 internações consecutivas por insuficiência cardíaca aguda em nosso departamento de cardiologia, todas submetidas a avaliação ecocardiográfica padronizada na admissão por operadores experientes. Entre 323 pacientes com fração de ejeção do ventrículo esquerdo (FEVE) > 40%, 252 apresentavam congestão sistêmica. Destes, 81,7% apresentavam doença valvar grave. Outros 10% apresentavam outras anormalidades importantes (por exemplo, miocardiopatias, hipertensão arterial pulmonar pré-capilar, distúrbios do ritmo hemodinamicamente relevantes, como arritmias ventriculares sustentadas e bloqueio atrioventricular avançado, ou doença renal em estágio V). Apenas 8% não apresentavam nenhuma causa estrutural ou extracardíaca identificável. Esses resultados, publicados na revista Internal and Emergency Medicine, publicação oficial da Sociedade Italiana de Medicina Interna, e posteriormente discutidos no European Journal of Internal Medicine, publicação oficial da Federação Europeia de Medicina Interna, foram comentados em inúmeras revisões e comentários publicados em periódicos internacionais,^2-7^ sugerem que a ICFEp verdadeiramente "pura" — foi definida pelas diretrizes como disfunção diastólica e aumento das pressões de enchimento, na ausência de outras causas primárias, como valvulopatia grave ou cardiomiopatia — é extremamente rara.

Esta observação alinha-se com a trajetória histórica da pesquisa sobre ICFEp. Ensaios clínicos iniciais, como o CHARM-Preserved,^8^ incluíram pacientes com FEVE preservada e sintomas inespecíficos, frequentemente sem avaliação ecocardiográfica abrangente (estrutural e do estado de fluidos) no momento da descompensação. Estudos ainda mais recentes, como TOPCAT,^9^ PARAGON-HF,^10^ DELIVER^11^ e EMPEROR-Preserved,^12^ tentaram refinar os critérios de inclusão, mas continuaram a depender fortemente de marcadores indiretos, como peptídeos natriuréticos e diagnósticos administrativos. É importante ressaltar que a ecocardiografia não foi realizada sistematicamente na admissão para confirmar anormalidades estruturais, avaliar congestão ou descartar imitações. Até mesmo os autores do PARAGON-HF reconheceram que alguns pacientes podem ter sido classificados incorretamente devido à falta de avaliação diagnóstica abrangente no início do estudo.^10^ Além disso, dadas as limitações da tecnologia de imagem no início dos anos 2000, é provável que muitos casos de doença valvar grave não tenham sido detectados. Ademais, a subutilização da ressonância magnética cardíaca ou da cintilografia nuclear limitou o reconhecimento da amiloidose transtirretina selvagem, uma condição cada vez mais reconhecida, e contribuiu para a criação da narrativa de "disfunção diastólica sem anormalidades estruturais significativas", que por sua vez moldou o conceito de ICFEp como uma síndrome distinta.

Nos últimos anos, o diagnóstico de ICpFE expandiu-se substancialmente, atingindo proporções epidêmicas. De fato, em enfermarias de medicina interna, a ICpFE é comumente diagnosticada em pacientes idosos que apresentam dispneia aguda, frequentemente no contexto de pneumonia ou exacerbação de DPOC (Doença Pulmonar Obstrutiva Crônica). Não raramente, um diagnóstico duplo (por exemplo, DPOC e ICpFE) é registrado apesar da ausência de avaliação ecocardiográfica na admissão que justifique o aumento das pressões de enchimento ou sobrecarga de volume. Nesse cenário, o diagnóstico de insuficiência cardíaca aguda é feito apenas após terapia diurética empírica em pacientes com dispneia aguda.

Neste contexto, a ICFEp se tornou um termo sindrômico aplicado com muita liberalidade em casos de incerteza diagnóstica, em vez de uma condição fisiopatológica bem definida.

Embora eu aprecie o esforço para refinar a terminologia excluindo imitadores, acredito que a mensagem mais urgente seja esta: a ICFEp, em sua forma "pura", é um constructo teórico raramente observado na prática clínica do mundo real. Para garantir a precisão diagnóstica e a adequação terapêutica, justifica-se o retorno a uma abordagem diagnóstica baseada na fisiologia, evitando a aplicação indiscriminada do rótulo de ICFEp.
