# Supplementary material for: A ICFEp Realmente Existe? Das Imitações à Classificação Errônea na Prática Clínica
Source: Arq Bras Cardiol. 2026 May 11;123(4):e20250379. [Article in Portuguese] doi: 10.36660/abc.20250379 (PMC13398811; doi:10.36660/abc.20250379)
Supplement: Translation

# Translation: Does HFpEF Really Exist? From Mimics to Misclassification in Clinical Practice

**DOI:** 10.36660/abc.20250379i

**Authors:** Maria Giulia Bellicini

**Affiliations:** 1 Brescia Italy ASST Spedali Civili di Brescia, Brescia – Italy

**Keywords:** Diastolic Heart Failure, Dyspnea, Heart Valve Diseases

Dear Editor,

I read with interest the recent article by Villacorta et al.^1^ addressing the distinction between "true" heart failure with preserved ejection fraction (HFpEF) and conditions that merely mimic it. Their effort to clarify this conceptual ambiguity is commendable and timely. However, I believe the issue extends further, even when HFpEF mimics such as amyloidosis, constrictive pericarditis, or severe valvular disease are carefully excluded, the clinical entity known as HFpEF often dissolves under closer scrutiny.

Over the past 18 months, I have conducted a retrospective analysis of 773 consecutive acute heart failure admissions to our cardiology department, all of whom underwent standardized echocardiographic assessment at admission by experienced operators. Among 323 patients with left ventricle ejection fraction (LVEF) >40%, systemic congestion was present in 252. Of these, 81.7% had severe valvular disease. An additional 10% had other major abnormalities (e.g., cardiomyopathies, precapillary pulmonary artery hypertension, hemodynamically relevant rhythm disorders such as ventricular sustained arrhythmias and advanced atrio-ventricular block, or stage V renal disease). Only 8% lacked any identifiable structural or extracardiac cause. These results, published in Internal and Emergency Medicine, the official journal of the Italian Society of Internal Medicine, and further discussed in the European Journal of Internal Medicine, the official journal of the European Federation of Internal Medicine, have been commented in numerous reviews and commetary published in international journals,^2-7^ suggest that truly guideline defined HFpEF — defined as diastolic dysfunction and increased filling pressures, in the absence of other primary causes such as severe valvular disease or cardiomyopathy — is exceedingly rare.

This observation aligns with the historical trajectory of HFpEF research. Early trials such as CHARM-Preserved^8^ enrolled patients based on preserved LVEF and nonspecific symptoms, often without comprehensive echocardiographic evaluation (structural and of fluid status) at the time of decompensation. Even more recent studies like TOPCAT,^9^ PARAGON-HF,^10^ DELIVER,^11^ and EMPEROR-Preserved^12^ attempted to refine inclusion criteria but continued to rely heavily on indirect markers like natriuretic peptides and administrative diagnoses. Importantly, echocardiography was not systematically performed at admission to confirm structural abnormalities, assess congestion, or rule out mimics. Even the authors of PARAGON-HF acknowledged that some patients may have been misclassified due to a lack of comprehensive diagnostic evaluation at baseline.^10^

Furthermore, given the limitations of imaging technology in the early 2000s, it is likely that many cases of severe valve disease were not detected. Moreover, the underuse of cardiac magnetic resonance imaging or nuclear scintigraphy limited the recognition of wild-type transthyretin amyloidosis, a condition now increasingly recognized, and contributed to the creation of the "diastolic dysfunction without significant structural abnormalities" narrative, which in turn shaped the HFpEF concept as a distinct syndrome.

In recent years, the diagnosis of HFpEF has expanded substantially, approaching epidemic proportions. Indeed in internal medicine wards, HFpEF is commonly assigned in elderly patients presenting with acute dyspnea, often in the context of pneumonia or COPD exacerbation. Not infrequently, a dual diagnosis (e.g., COPD and HFpEF) is recorded despite the absence of echocardiographic assessment at admission to support elevated filling pressures or volume overload. Inthis scenario acute haert failure diagnosis is made only ex adiuvantibus following empirical diuretic therapy in patients with acute dyspnoea.

In this context HFpEF has become a syndromic label applied too liberally in cases of diagnostic uncertainty, rather than a well-defined pathophysiologic condition.

While refining the terminology by excluding mimics is valuable, the more pressing message is that guideline-defined HFpEF represents a theoretical construct that is rarely encountered in real-world clinical practice. To ensure diagnostic accuracy and appropriate therapy, a return to a physiology-based diagnostic framework is warranted, avoiding the indiscriminate use of the HFpEF label.
