# Supplementary material for: A ICFEp Realmente Existe? Das Imitações à Classificação Errônea na Prática Clínica
Source: Arq Bras Cardiol. 2026 May 11;123(4):e20250379. [Article in Portuguese] doi: 10.36660/abc.20250379 (PMC13398811; doi:10.36660/abc.20250379)
Supplement: Related texts

# Reply: Carta-resposta

**Authors:** Humberto Villacorta, Pedro Schwartzmann

**Affiliations:** 1 Universidade Federal Fluminense Niterói RJ Brasil Universidade Federal Fluminense, Niterói, RJ – Brasil; 2 Universidade de Sao Paulo Campus de Ribeirão Preto Ribeirão Preto SP Brasil Universidade de Sao Paulo Campus de Ribeirão Preto, Ribeirão Preto, SP – Brasil

Caro Editor,

Agradecemos ao autor da carta por se envolver cuidadosamente com nosso artigo e por compartilhar insights sobre uma síndrome complexa como a ICFEp. Nosso artigo se concentrou apenas na distinção entre ICFEp e seus imitadores, e muitos aspectos apontados pelos autores não estavam dentro do escopo do nosso manuscrito. No entanto, é importante esclarecer alguns pontos levantados pelos autores. Embora concordemos com algumas observações apresentadas, discordamos respeitosamente da conclusão de que a ICFEp é, em grande parte, um construto teórico.

Concordamos que a ICFEp é frequentemente superdiagnosticada em ambientes de cuidados agudos, especialmente na ausência de exames de imagem e de avaliação hemodinâmica robustos. A observação do autor de que muitos pacientes com ICFEp apresentam anormalidades estruturais significativas, como doença valvar grave, distúrbios do ritmo ou fatores extracardíacos contribuintes, como DPOC e insuficiência renal, é consistente com nossa própria posição: esses pacientes não têm ICFEp. Trata-se de casos de diagnóstico incorreto.

No entanto, a afirmação de que a ICFEp "pura" é "extremamente rara" ou mesmo uma entidade "irreal" exige um exame mais detalhado. Em primeiro lugar, a definição de ICFEp evoluiu substancialmente nas últimas duas décadas. As diretrizes contemporâneas agora enfatizam a avaliação abrangente, incluindo evidências de disfunção diastólica, níveis elevados de peptídeos natriuréticos, sinais de doença cardíaca estrutural (p. ex., aumento do átrio esquerdo, hipertrofia do ventrículo esquerdo) e pressões de enchimento elevadas. Portanto, a afirmação de que a ICFEp é definida como "descompensação aguda em um coração estruturalmente normal" está incorreta. É exatamente o oposto. Precisamos demonstrar que o coração foi afetado usando o biomarcador cardíaco padrão-ouro para o diagnóstico de insuficiência cardíaca – peptídeos natriuréticos – e/ou a demonstração de alterações estruturais do coração, como mencionado acima. Por favor, observe que a definição de ICFEp inclui uma FEVE normal, mas não um coração estruturalmente normal. Esses conceitos estão bem demonstrados na definição universal mais recente de insuficiência cardíaca.^1^

Também alertamos contra a definição dos autores como "descompensação aguda…". A ICFEp é frequentemente uma condição crônica com exacerbações intermitentes desencadeadas por comorbidades. Na verdade, a maioria dos pacientes se apresenta em regime ambulatorial, com queixa de intolerância ao exercício.

Embora os ensaios iniciais possam ter incluído pacientes com critérios menos rigorosos, estudos recentes como EMPEROR-Preserved,^2^ DELIVER^3^ e FINEARTS-HF^4^ refinaram significativamente a seleção de pacientes. A inclusão de pacientes com doença cardíaca valvar nesses ensaios é bastante improvável, visto que um ecocardiograma era obrigatório para confirmar uma FEVE > 50% (ou > 40% em alguns estudos, incluindo também aqueles com FEVE levemente reduzida), o que faz parte da própria definição de ICFEp.

O autor destaca, com razão, o subdiagnóstico de condições como a amiloidose por transtirretina no passado. No entanto, o aumento do rendimento diagnóstico da ressonância magnética cardíaca, da imagem nuclear e do perfil de biomarcadores apenas reforçou a distinção entre ICFEp e outras doenças infiltrativas ou valvares – e não a obscureceu.

A ICFEp abrange uma síndrome fisiopatológica distinta, marcada por inflamação sistêmica, enrijecimento cardíaco, disfunção endotelial, incompetência cronotrópica e acoplamento ventrículo-arterial anormal — frequentemente coexistindo com comorbidades como hipertensão, obesidade e diabetes. Essas alterações não são meramente fatores de confusão, mas participantes ativos na manifestação da doença. Essas alterações resultam em um VE rígido, como demonstrado por Zile et al. em 2004.^5^ A rigidez do VE leva ao aumento da pressão de enchimento e ao aumento do volume atrial esquerdo. Sua expressão clínica é heterogênea por natureza, não sendo uma falha conceitual.

Concluindo, acolhemos o apelo por rigor diagnóstico e pensamento fisiológico. No entanto, em vez de invalidar a ICFEp, essa perspectiva reforça a necessidade de fenotipagem precisa, exclusão cuidadosa de imitadores e aprimoramento contínuo das vias diagnósticas. A ICFEp existe – não como uma doença específica, mas como um espectro de doenças enraizadas em mecanismos identificáveis que merecem terapia adequada.

## References

[1] Bozkurt B, Coats AJ, Tsutsui H, Abdelhamid M, Adamopoulos S, Albert N (2021). Universal Definition and Classification of Heart Failure: A Report of the Heart Failure Society of America, Heart Failure Association of the European Society of Cardiology, Japanese Heart Failure Society and Writing Committee of the Universal Definition of Heart Failure. J Card Fail.

[2] Anker SD, Butler J, Filippatos G, Ferreira JP, Bocchi E, Böhm M (2021). Empagliflozin in Heart Failure with a Preserved Ejection Fraction. N Engl J Med.

[3] Solomon SD, McMurray JJV, Claggett B, de Boer RA, DeMets D, Hernandez AF (2022). Dapagliflozin in Heart Failure with Mildly Reduced or Preserved Ejection Fraction. N Engl J Med.

[4] Solomon SD, McMurray JJV, Vaduganathan M, Claggett B, Jhund PS, Desai AS (2024). Finerenone in Heart Failure with Mildly Reduced or Preserved Ejection Fraction. N Engl J Med.

[5] Zile MR, Baicu CF, Gaasch WH (2004). Diastolic Heart Failure--Abnormalities in Active Relaxation and Passive Stiffness of the Left Ventricle. N Engl J Med.

# Reply

**Authors:** Humberto Villacorta, Pedro Schwartzmann

**Affiliations:** 1 Niterói RJ Brazil Universidade Federal Fluminense, Niterói, J – Brazil; 2 Ribeirão Preto SP Brazil Universidade de Sao Paulo Campus de Ribeirão Preto, Ribeirão Preto, SP – Brazil

Dear Editor,

We thank the author of the letter for engaging thoughtfully with our article and for sharing insights on a complex syndrome such as HFpEF. Our paper focused only on the distinction between HFpEF and its mimics, and many aspects pointed out by the authors were not within the scope of our manuscript. However, it is important to clarify some points brought up by the authors. While we agree with some observations presented, we respectfully disagree with the conclusion that HFpEF is largely a theoretical construct.

We agree that HFpEF is frequently overdiagnosed in acute care settings, especially in the absence of robust imaging and hemodynamic assessment. The author's observation that many patients labeled as HFpEF exhibit significant structural abnormalities, such as severe valvular disease, rhythm disorders, or extracardiac contributors like COPD and renal failure, is consistent with our own position: these patients do not have HFpEF. These are cases of misdiagnosis.

However, the assertion that "pure" HFpEF is "exceedingly rare" or even an "unreal" entity demands closer scrutiny. First, the definition of HFpEF has evolved substantially over the past two decades. Contemporary guidelines now emphasize comprehensive evaluation, including evidence of diastolic dysfunction, elevated natriuretic peptides, signs of structural heart disease (e.g., left atrial enlargement, LV hypertrophy), and elevated filling pressures. So, the statement that HFpEF is defined as "acute decompensation in a structurally normal heart" is incorrect. It is quite the opposite. We need to demonstrate that the heart has been affected either using the gold-standard cardiac biomarker for the diagnosis of heart failure – natriuretic peptides – and/or the demonstration of structural alterations of the heart, as mentioned above. Please observe that the definition of HFpEF includes a normal LVEF but not a structurally normal heart. These concepts are well demonstrated in the latest universal definition of heart failure.^1^

We also caution against the definition by the authors as an "acute decompensation…". HFpEF is frequently a chronic condition with intermittent exacerbations triggered by comorbidities. Actually, most of the patients present as ambulatory cases, with a complain of exercise intolerance.

While early trials may have included patients with less stringent criteria, recent studies such as EMPEROR-Preserved,^2^ DELIVER,^3^ and FINEARTS-HF^4^ have significantly refined patient selection. The inclusion of patients with valvular heart disease in these trials is quite unlikely since an echocardiogram was mandatory to confirm a LVEF ≥50% (or ≥40% in some studies, to include those with mildly reduced LVEF as well), which is part of the definition of HFpEF itself.

The author rightly highlights the under-recognition of conditions like transthyretin amyloidosis in the past. Nevertheless, the increasing diagnostic yield of cardiac magnetic resonance imaging, nuclear imaging, and biomarker profiling has only strengthened the distinction between HFpEF and other infiltrative or valvular diseases – not blurred it.

HFpEF encompasses a distinct pathophysiological syndrome marked by systemic inflammation, cardiac stiffening, endothelial dysfunction, chronotropic incompetence, and abnormal ventricular-arterial coupling—often coexisting with comorbidities such as hypertension, obesity, and diabetes. These are not merely confounders, but active participants in disease manifestation. These alterations result in a stiff LV, as demonstrated by Zile et al. in 2004.^5^ The stiffness of the LV leads to increased filling pressure and increased left atrial volume. Its clinical expression is heterogeneous by nature, not a flaw in the concept.

In conclusion, we welcome the call for diagnostic rigor and physiological thinking. However, rather than invalidating HFpEF, this perspective reinforces the need for precise phenotyping, careful exclusion of mimics, and ongoing refinement of diagnostic pathways. HFpEF does exist – not as a specific disease, but as a spectrum of diseases rooted in identifiable mechanisms deserving proper therapy.
